# Differences in joint morphology between the knee and ankle affect the repair of osteochondral defects in a rabbit model

**DOI:** 10.1186/s13018-016-0444-4

**Published:** 2016-10-04

**Authors:** Manami Makitsubo, Nobuo Adachi, Tomoyuki Nakasa, Tomohiro Kato, Ryo Shimizu, Mitsuo Ochi

**Affiliations:** Department of Orthopaedic Surgery, Integrated Health Sciences, Institute of Biomedical and Health Sciences, Hiroshima University, 1-2-3 Kasumi, Minami-ku, Hiroshima 734-8551 Japan

**Keywords:** Ankle, Knee, Congruency, Subchondral bone, Rabbit

## Abstract

**Background:**

Although differences in the results of the bone marrow stimulation technique between the knee and ankle have been reported, a detailed mechanism for those differences has not been clarified. The purpose of this study was to examine whether morphological differences between the knee and ankle joint affect the results of drilling as treatment for osteochondral defects in a rabbit model.

**Methods:**

Osteochondral defects were created at the knee and ankle joint in the rabbit. In the knee, osteochondral defects were created at the medial femoral condyle (MFC) and patellar groove (PG). At the ankle, defects were created in the talus at either a covered or uncovered area by the tibial plafond. After creating the osteochondral defect, drilling was performed. At 4, 8, and 12 weeks after surgery, repair of the osteochondral defects were evaluated histologically. The proliferation of rabbit chondrocytes and proteoglycan release of cartilage tissue in response to IL-1β were analyzed in vitro in both joints.

**Results:**

At 8 weeks after surgery, hyaline cartilage repair was observed in defects at the covered area of the talus and the MFC. At 12 weeks, hyaline cartilage with a normal thickness was observed for the defect at the covered area of the talus, but not for the defect at the MFC. At 12 weeks, subchondral bone formation progressed and a normal contour of subchondral bone was observed on CT in the defect at the covered area of the talus. No significant differences in chondrocyte proliferation rate and proteoglycan release were detected between the knee and ankle in vitro.

**Conclusions:**

Our results demonstrate that the covered areas of the talus show early and sufficient osteochondral repair compared to that of the knee and the uncovered areas of the talus. These results suggest that the congruent joint shows better subchondral repair prior to cartilage repair compared to that of the incongruent joint.

## Background

Articular cartilage has a limited ability of repair, and untreated lesions of articular cartilage may progress to osteoarthritis (OA) [1, 2]. Many strategies for the treatment of articular cartilage have been developed including bone marrow stimulation techniques [3, 4], osteochondral grafts [5], and tissue engineering approaches [1]. Articular cartilage consists of sparse chondrocytes and dense extracellular matrix of mainly type 2 collagen and proteoglycans. Articular cartilage lacks blood vessels and nervous innervation, which makes the repair of articular cartilage difficult [6]. The constitution of the cartilage differs between joints in individuals, which may result in differences in the results of treatment and the progression of OA. Several reports have demonstrated constitutional and biochemical differences of articular cartilage between the knee and ankle [7, 8].

For the repair of articular cartilage, bone marrow stimulation techniques have been performed widely. Bone marrow stimulation creates blood clots in the area of the defect through methods such as microfracture or drilling. Results after drilling of the talus have reported good outcome in 80–96 % of cases [9–11]. On the other hand, results after drilling of the medial femoral condyle (MFC) have reported good outcome in 69–80 % of cases [12–14]. As a whole, the results of the talus are better than those of the MFC.

Factors influencing differences in results after bone marrow stimulation between the knee and ankle have not been explored. As suggested in previous reports, biochemical and constitutional differences between the knee and ankle cartilages may affect the capacity of the cartilage to repair after bone marrow stimulation [15]. However, differences in the joint morphology between the knee and ankle should be examined. As for the knee joint, the femoral condyle and the tibia plateau do not have good congruency, while the ankle joint has good congruency as a tenon and mortise structure. This difference in joint morphology may affect the capacity of cartilage repair. OA is induced by cartilage injury, and the incidence of primary OA is different between the knee and ankle. Symptomatic OA with radiographical signs occurs about eight to nine times more frequently in the knee than in the ankle joint [16, 17]. OA induced by cartilage injury may progress due to the degeneration of adjacent cartilage, and catabolic factors, such as inflammatory cytokines and biomechanical stress, in the cartilage around the defect may affect the progression of OA after cartilage injury. There may be differences in the chondrocyte response to inflammatory cytokines and proliferation between the knee and ankle, which may lead to a difference in the progression of OA after cartilage injury between the knee and ankle joint. Understanding the factors that may contribute to differences in the outcome of cartilage repair and incidence of OA between knee and ankle may improve outcome after treatment.

We hypothesize that the repair of articular cartilage in the congruent joint is better than that in the non-congruent joint. The purpose of this study was to examine differences in cartilage repair after drilling between the talus (congruent joint) and knee (non-congruent joint) in a rabbit model. In addition, differences between the knee and ankle as to the proliferation ability of chondrocytes, the production of proteoglycans (patellar groove (PG)), and the reaction of cartilage to IL-1β were also examined in vitro*.* Together, results may clarify the roles of morphological and biochemical factors in differences in cartilage degeneration between the knee and ankle.

## Methods

Rabbits were housed in the research facilities for laboratory animal science. The experimental research protocol was reviewed and approved by the Hiroshima University ethical committee.

### Surgical procedure

**Fig. 1:**
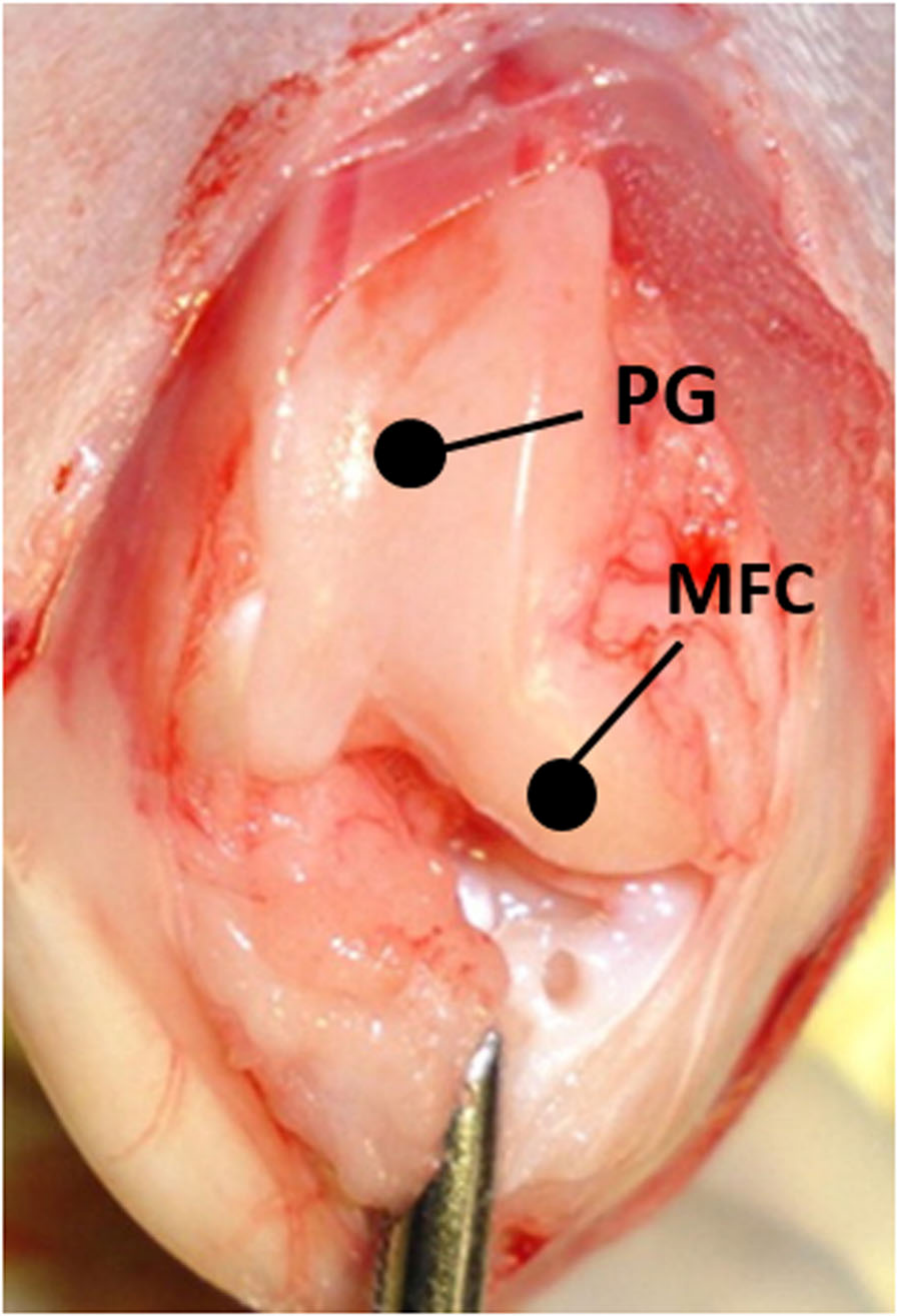
Osteochondral defect sites at the knee. *PG* patellar groove, *MFC* medial femoral condyle

**Fig. 2:**
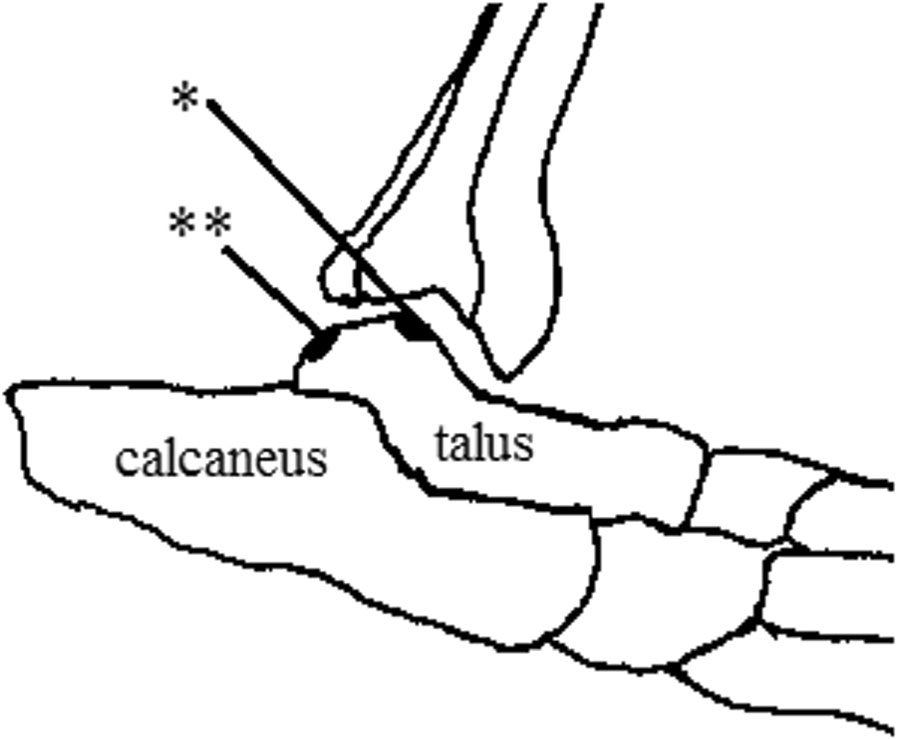
Osteochondral defect sites at the talus (schematic illustration). *Single asterisk*, covered talus; *double asterisk*, uncovered talus

Eighteen male Japanese white rabbits (3.0–3.5 kg; Kitayama Labs, Nagano Japan) were used. The rabbits were anesthetized by intravenous injection of pentobarbital (30 mg/kg) supplemented with subcutaneous injection of 1 % xylocaine. The knees and ankles were depilated and disinfected with 70 % alcohol. Osteochondral defects were created at the MFC of the left knee, PG of the right knee, and bilateral tali. For the knee joint, the patella was dislocated laterally through a medial parapatellar approach, and the osteochondral defect was created at the MFC or PG. The defect site of the MFC was created at the center and tip of the MFC, a partially weight-bearing area. The weight-bearing area in the flexed knee of rabbits is at the inferoposterior aspect [18]. The osteochondral defect of the patellar groove was created at the center of the groove and under the patella in a flexed position (Fig. 1). Two types of osteochondral defects were created at the talus (Fig. 2). The osteochondral defect at the center of the left talus was defined as a covered area (covered talus) that contacts the articular surface of the plafond of the tibia during all motion of the ankle joint. The osteochondral defect at the posterior of the cartilage area of the right talus was defined as an uncovered area (uncovered talus). In this area, the talus hardly contacts the surface of the plafond because the ankle joint of the caged rabbits is in dorsiflexion most of the time. For the left talus, a straight skin incision was applied at the anterior of the joint. After the extensor retinaculum was incised, arthrotomy was performed and the osteochondral defect of the talus was created. The extensor retinaculum was repaired. For the right talus, a straight skin incision was applied medial to the Achilles tendon. The Achilles tendon was dislocated laterally, and the osteochondral defect was created at the posterior of the talus.

The osteochondral defects (3.0 mm in diameter and 2.0 mm in depth) were created using a punch. Drilling was performed at four points using a 0.7-mm Kirschner wire (K-wire) as described previously [19].

### Micro-CT and histological evaluation

The animals were sacrificed at 4, 8, and 12 weeks after surgery with an overdose of sodium pentobarbital. The knee and ankle joints were dissected from the muscle. Distal portions of the femur and talus were removed and fixed in 10 % phosphate-buffered formalin (pH 7.0). Micro-CT (Skyscan X-ray Microtomography 1172, Konitich, Belgium) was performed to evaluate the defect repair. The distal end of the left femur (MFC) was cut in the sagittal plane, the right femur (PG) was cut in the axial plane, and both ankles (tali) were cut in the coronal plane. After decalcification in 10 % EDTA (Nacalai Tesque, Inc., Kyoto, Japan) and paraffin embedding, the sections (4-μm thick) were cut perpendicular to the joint surface and stained with Safranin O fast green. Histological evaluation was performed using the Pineda score under a light microscope (Table 1) [20]. Histological grading was performed by two observers who were not aware of the source of the samples.

**Table 1:** Pineda histologic score for cartilage repair [20]

Filling of defect (%)	
125	1
100	0
75	1
50	2
25	3
0	4
Reconstruction of osteochondral junction	
Yes	0
Almost	1
Not close	2
Matrix staining	
Normal	0
Reduced staining	1
Significantly reduced staining	2
Faint staining	3
No staining	4
Cell morphology	
Normal	0
Mostly hyaline and fibrocartilage	1
Mostly fibrocartilage	2
Some fibrocartilage, but mostly	
Non-chondrocytic cells	3
Non-chondrocytic cells only	4

The score ranged from 0 (normal) to 14 (worst)

### Immunohistochemical evaluation

The sections at 12 weeks were pretreated with antigen retrieval reagent (Immunoactive, Matsunami Glass Ind., Osaka, Japan) for 1 h followed by 0.3 % H_2_O_2_ for 30 min, normal blocking serum for 30 min, and primary antibody against type 2 collagen (dilution, 1:100; anti-hCL(II); Daiichi Fine Chemical, Toyama, Japan), type 1 collagen (dilution, 1:250; Novus Biologicals, USA), and MMP13 (dilution, 1:15; Neo Markers, CA, USA) overnight at 4 °C. The next day, the sections were visualized using the avidin-biotin system (Vectastain Elite ABC Mouse IgG kit, Vector Laboratories, Inc., Burlingame, CA) and 3,3′diaminobenzidine (Peroxidase Substrate Kit, Vector Laboratories, Inc.) according to the manufacturer’s instructions.

To quantitate the immunochemistry results, the number of immune-positive cells in each type of osteochondral defect was counted in a microscopic field (100 μm × 100 μm) under ×40 magnification.

### Proliferation ability of chondrocytes

Cartilage tissue was obtained from the distal femur and talus. Chondrocytes were isolated from the cartilage tissues and cultured. The tissues were minced and incubated in trypsin (Tryp LE Express; Life Technologies, Carlsbad, CA) for 15 min at 37 °C, after which the cartilage was treated with Dulbecco’s modified Eagle’s medium (DMEM; Gibco BR, Grand Island, NY, USA) containing 0.2 % collagenase (Sigma, St. Louis, MO) at 37 °C for 4 h. Dissociated cells were cultured in DMEM supplemented with 10 % fetal bovine serum (FBS; Biowhittaker, Walkersville, MD, USA) and 100 U/ml of penicillin-streptomycin. After overnight culture, non-adherent cells were removed, and adherent cells were further incubated in fresh medium. Chondrocytes were seeded in 96-well plates at a density of 5 × 10^3^ cells per well in DMEM medium supplemented with 10 % FBS. At 4 h after cell plating, cell proliferation was assessed using a Cell Counting Kit-8 (Dojindo, Kumamoto, Japan) (4-[3-(4-iodophenyl)-2-(4-nitrophenyl)-2H-5-tetrazolio]-1,3-benzene disulfonate (WST) assay) at day 1 according to the manufacturer’s instruction. The values, corresponding to the number of viable cells, were read at OD 450 mm using a Microplate Reader (BioTek Instrument, Winooski, VT, USA). The cell increase ratios at 2 and 3 days were compared between the femur and talus.

### Production ability of proteoglycans and the reaction to IL-1β

The cartilage tissue was obtained from the distal femur and talar domes. Samples are incubated at 37 °C for 72 h in 48-well plates. Each well contained 500 μl of DMEM with 10 % FBS and 1 % penicillin/streptomycin. The cartilage samples were washed three times and cultured at 37 °C for an additional 72 h in 500 μl of serum-free DMEM with IL-1β (1 ng/ml; Peprotech, Rocky Hill, NJ, USA) or without IL-1β. The assay was performed in at least three independent experiments with duplicate wells. The concentration of the released glycosaminoglycan in the cartilage-conditioned medium was determined using the Blyscan Glycosaminoglycan assay kit (Biocolor, UK) according to the manufacturer’s protocol. Proteoglycan release quantity with IL-1β/without IL-1β ratio was compared between the femur and talus.

### Statistical analysis

The Kruskal-Wallis test was used to analyze the histological scoring data and quantitative values of immunochemistry among the groups. If a significant difference was obtained, the Steel-Dwass test was used to perform multiple comparisons between the groups. In vitro data were analyzed using the Mann-Whitney *U* test to determine significant differences between the femur and talus. A *P* value of <0.05 was considered significant.

## Results

### Histological evaluation

**Fig. 3:**
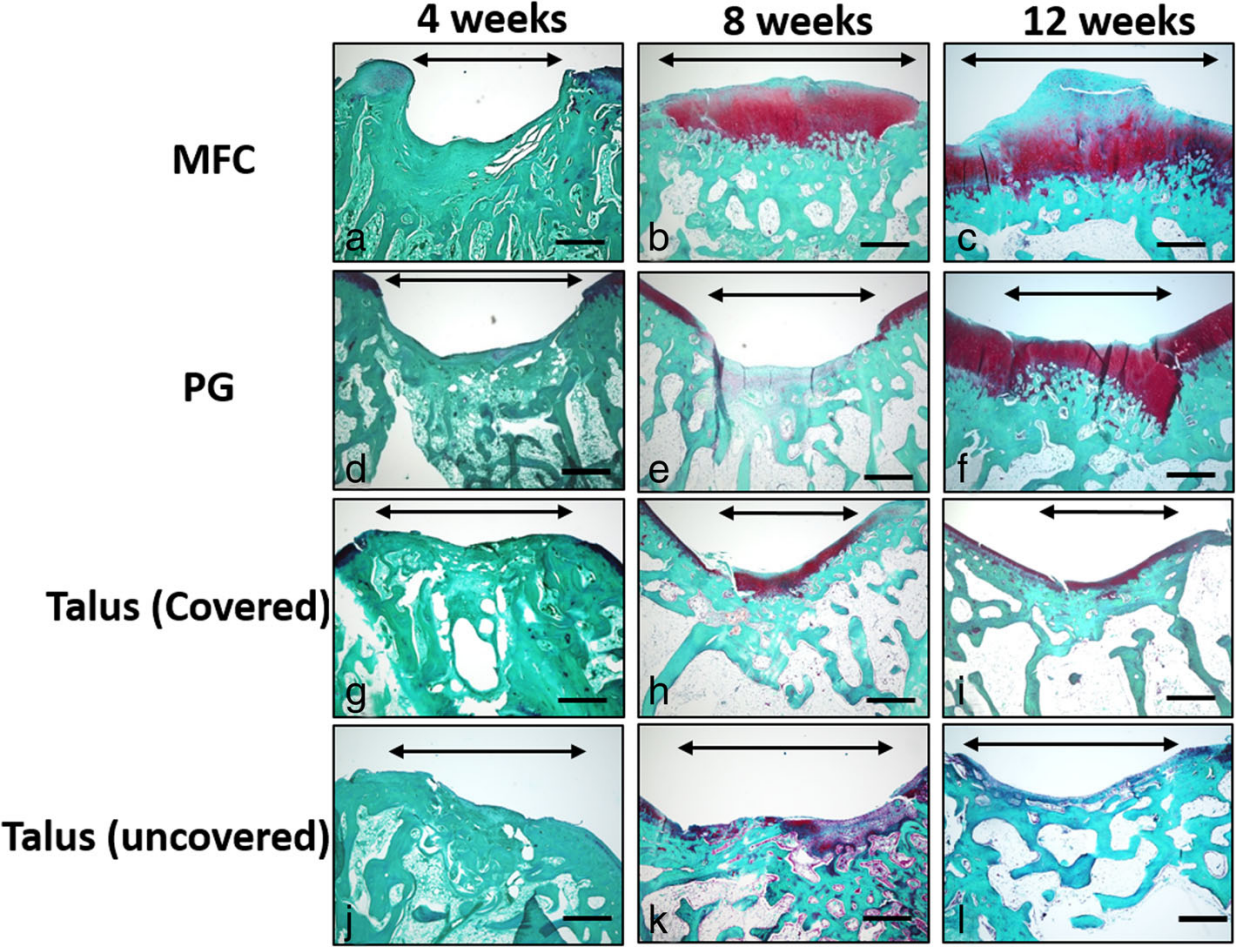
Histological findings of the osteochondral defect of the medial femoral condyle (MFC) (**a**–**c**), patellar groove (PG) (**d**–**f**), covered talus (**g**–**i**), and uncovered talus (**j**–**l**) at 4, 8, and 12 weeks after surgery. *Bidirectional arrows* indicate the osteochondral defect. *Scale bar*, 500 μm

At 4 weeks after surgery, osteochondral defect was observed in the MFC and PG with a small amount of fibrous tissue (Fig. 3a, d). In the covered and uncovered talus, partial subchondral bone repair was observed and the defect was filled with fibrous tissue (Fig. 3g, j). In the MFC at 8 weeks, hyaline cartilage repair was observed, but subchondral bone repair was delayed, with cartilage observed at subchondral bone area (Fig. 3b). In the PG, hyaline cartilage repair was not observed (Fig. 3e). In the covered talus, hyaline cartilage repair was observed and cartilage at the site of the defect was thicker than the adjacent normal cartilage (Fig. 3h). In the uncovered talus, hyaline cartilage repair was hardly observed (Fig. 3k). In the MFC at 12 weeks, the cartilage layer was thickened, but subchondral bone repair was present (Fig. 3c). In the PG, hyaline cartilage repair was observed, but the subchondral bone plate repair was delayed and cartilage was observed on the subchondral plate (Fig. 3f). In the covered and uncovered talus at 12 weeks, the thickness of the cartilage at the defect was similar to that of the adjacent cartilage (Fig. 3i, l). Thus, hyaline cartilage repair was observed in the covered talus, but not in the uncovered talus.

**Fig. 4:**
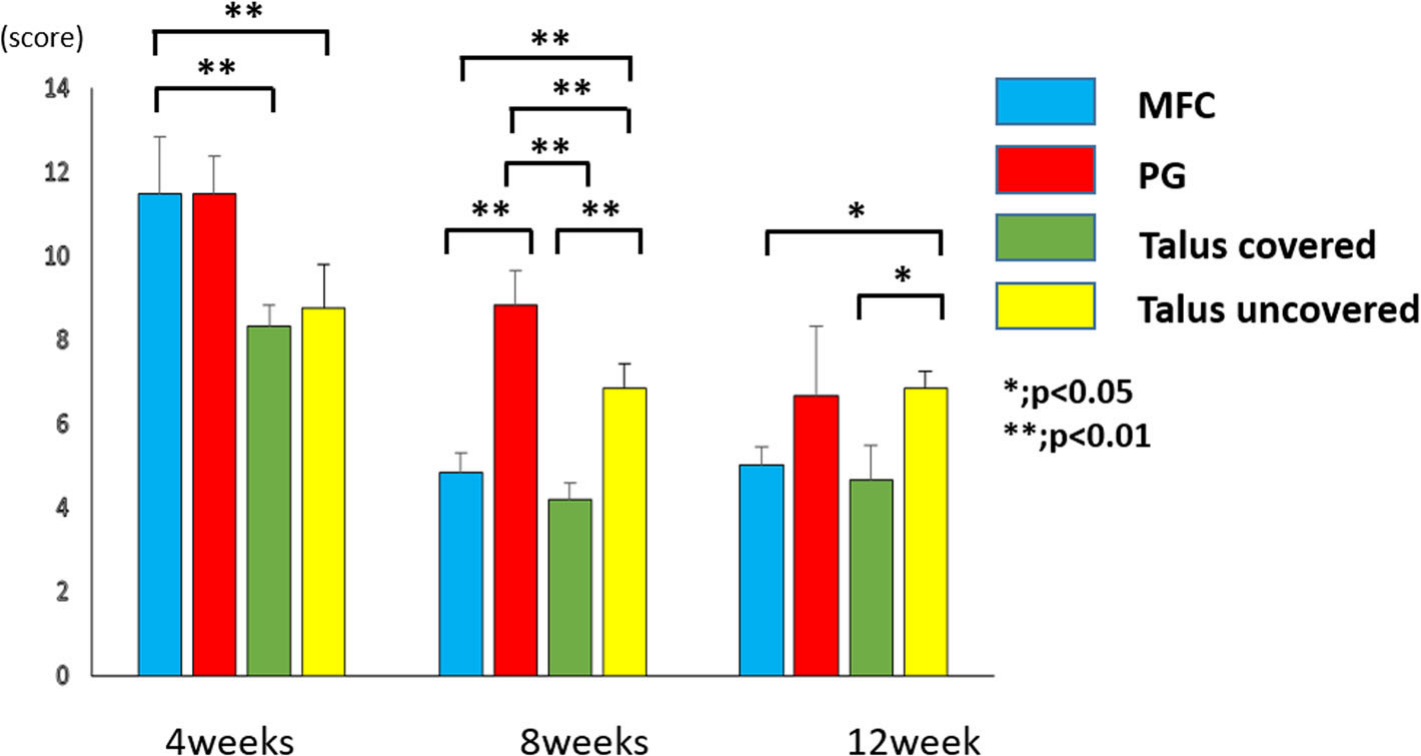
Results of the Pineda score. *P < 0.05; **P < 0.01. *N* = 6 for each groups

Pineda scores are shown in Fig. 4. At 4 weeks, the covered and uncovered talus showed significantly better scores than the MFC (*P* < 0.01). At 8 weeks, the covered talus and the MFC showed significantly better scores than the PG and the uncovered talus (*P* < 0.01). There was a significant difference between the PG and the uncovered talus (*P* < 0.01). At 12 weeks, the difference in the Pineda score between the PG and the uncovered talus was reduced. However, the covered talus and the MFC scored significantly better than the uncovered talus (*P* < 0.05).

### Micro-CT evaluation

**Fig. 5:**
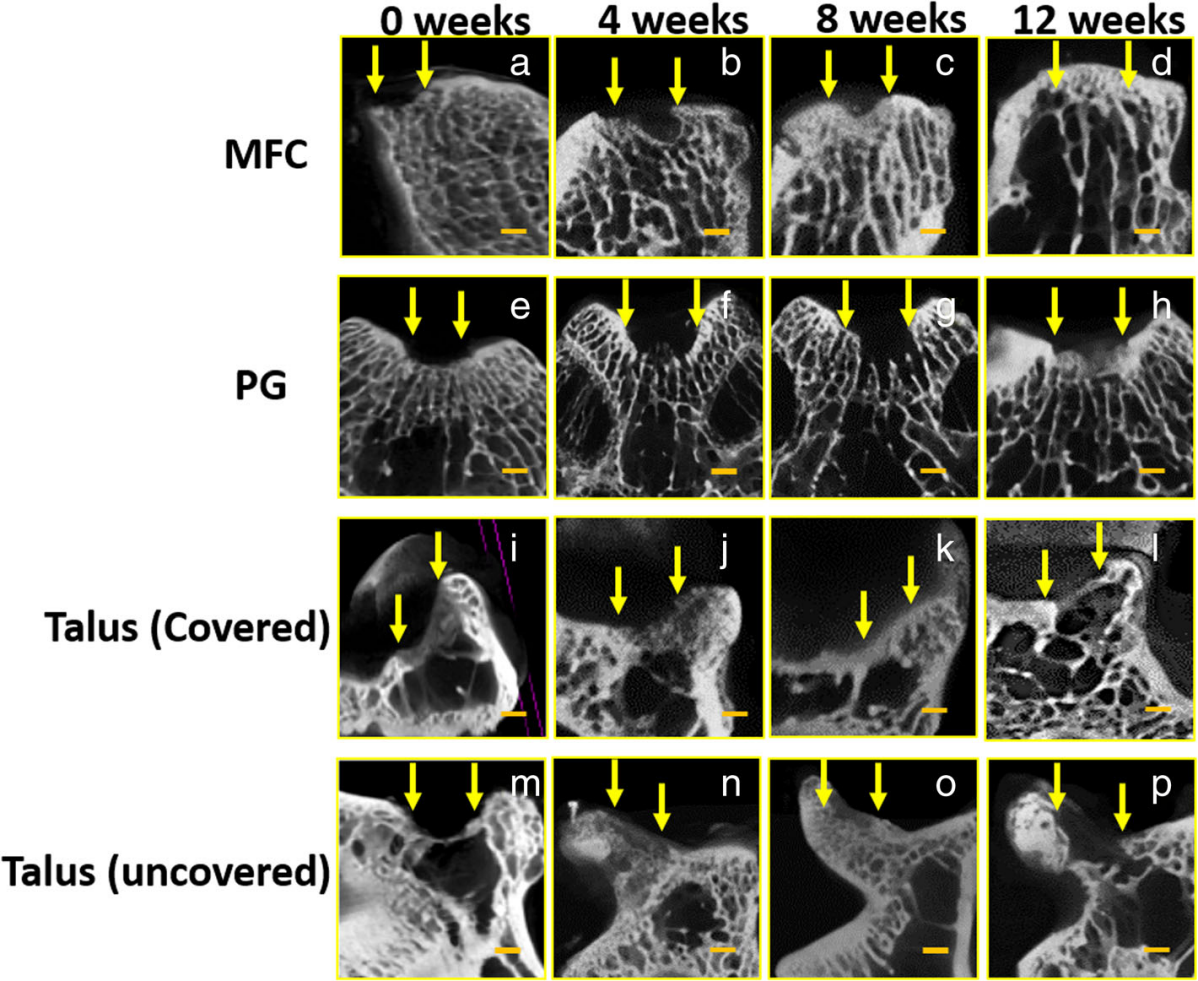
Computed tomography findings of osteochondral defects of the medial femoral condyle (MFC) (**a**–**d**), patellar groove (PG) (**e**–**h**), covered talus (**i**–**l**), and uncovered talus (**m**–**p**) at 0, 4, 8, and 12 weeks after surgery. *Arrows* indicate the osteochondral defect. *Scale bar*, 1000 μm

On micro-CT evaluation, subchondral bone formation was observed at 4 weeks in the covered talus (Fig. 5j). In the uncovered talus, two samples showed subchondral formation at 4 weeks (Fig. 5n), while no subchondral formation was observed in the MFC and PG (Fig. 5b, f). At 8 weeks, the covered talus showed progress in the remodeling of the subchondral plate, and the subchondral plate at the defect was at a similar level as the adjacent subchondral bone plate (Fig. 5k). Progression of remodeling of the subchondral plate was also observed in the uncovered talus in three samples (Fig. 5o). In the MFC, bone formation of the subchondral plate was progressed in two samples (Fig. 5c), but no sample showed progress in bone formation of the subchondral plate in the PG (Fig. 5g). At 12 weeks, in the covered talus, subchondral bone formation progressed, and a normal contour of subchondral bone was observed (Fig. 5l). In the uncovered talus, subchondral bone formation progressed, but the level of adjacent subchondral bone plate was irregular (Fig. 5p). In the MFC, good subchondral bone formation and contour was observed in all but one sample (Fig. 5d). In the PG, only one sample showed subchondral bone plate formation (Fig. 5h).

### Immunohistochemical evaluation

**Fig. 6:**
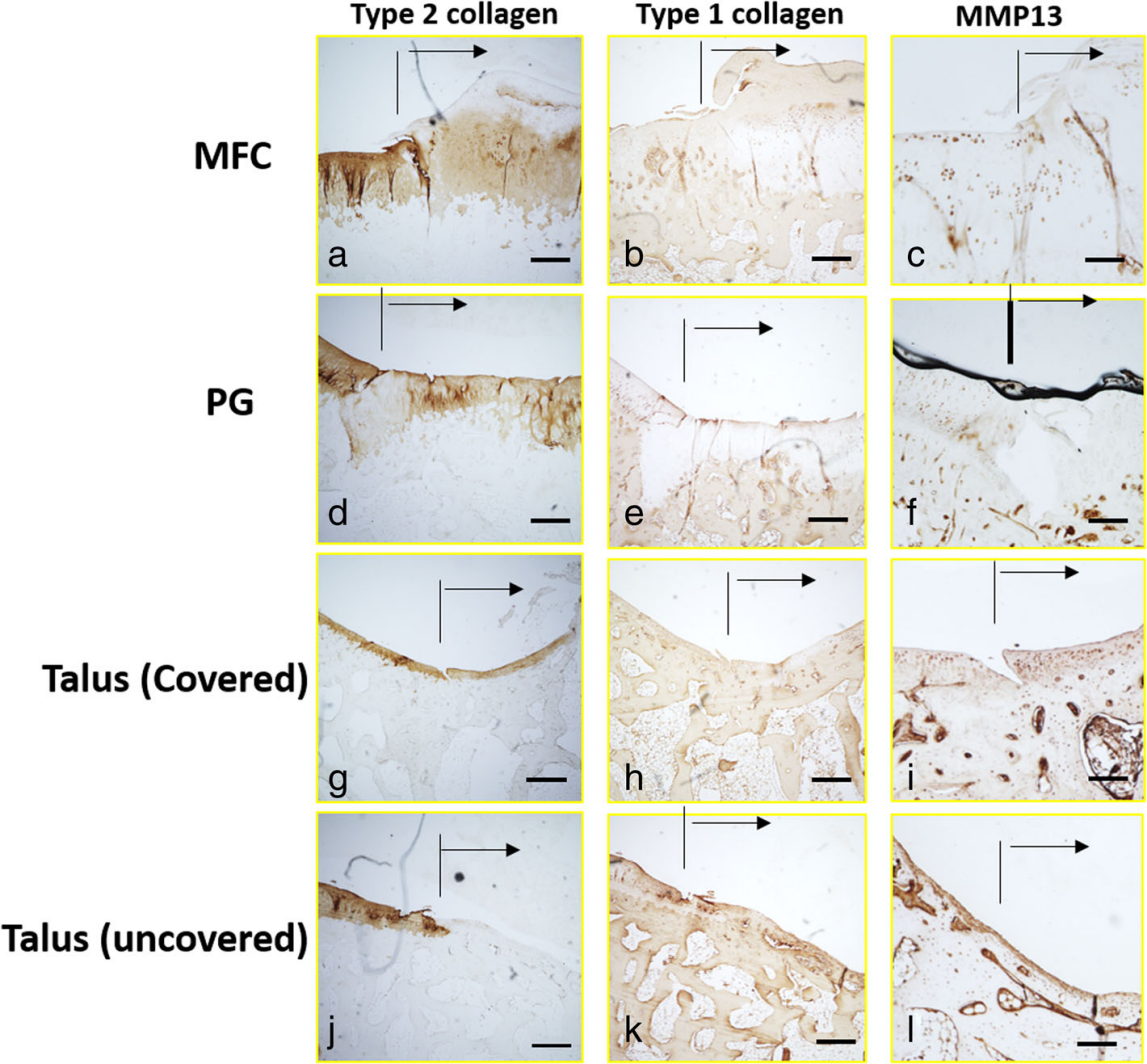
Immunohistochemistry of type 2 collagen, type 1 collagen, and MMP13 in the osteochondral defect of the medial femoral condyle (MFC) (**a**–**c**), patellar groove (PG) (**d**–**f**), covered talus (**g**–**i**), and uncovered talus (**j**–**l**) at 12 weeks after surgery. *Arrows* indicate the osteochondral defect. *Scale bar*, 500 μm

**Fig. 7:**
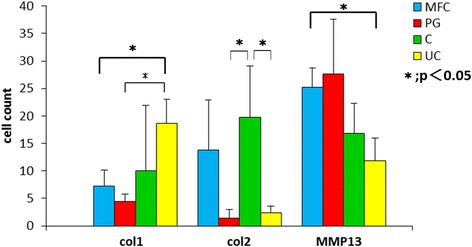
Results of quantitative values of immunochemistry of type 2 collagen, type 1 collagen, and MMP13 in the osteochondral defect of the medial femoral condyle (MFC), patellar groove (PG), covered talus (C), and uncovered talus (UC). *P < 0.05. *N* = 5 for each groups

The repair tissue in the covered talus showed the highest immunoreactivity for type 2 collagen of the samples, and in the MFC, moderate positive immunoreactivity was observed. In the PG and uncovered talus, no immunoreactivity was observed. There was a significant difference between the covered talus and PG and the covered talus and uncovered talus (*P* < 0.05). Immunoreactivity for type 1 collagen was faint in the MFC, PG, and covered talus. The uncovered talus showed only positive staining. The uncovered talus showed significantly more immunoreactive cells than the MFC and PG (*P* < 0.05). Immunoreactivity for MMP13 was higher in the MFC and PG. The covered talus and uncovered talus showed moderate positive immunoreactivity. There was a significant difference between the MFC and uncovered talus (Figs. 6 and 7).

### Proliferation ability

**Fig. 8:**
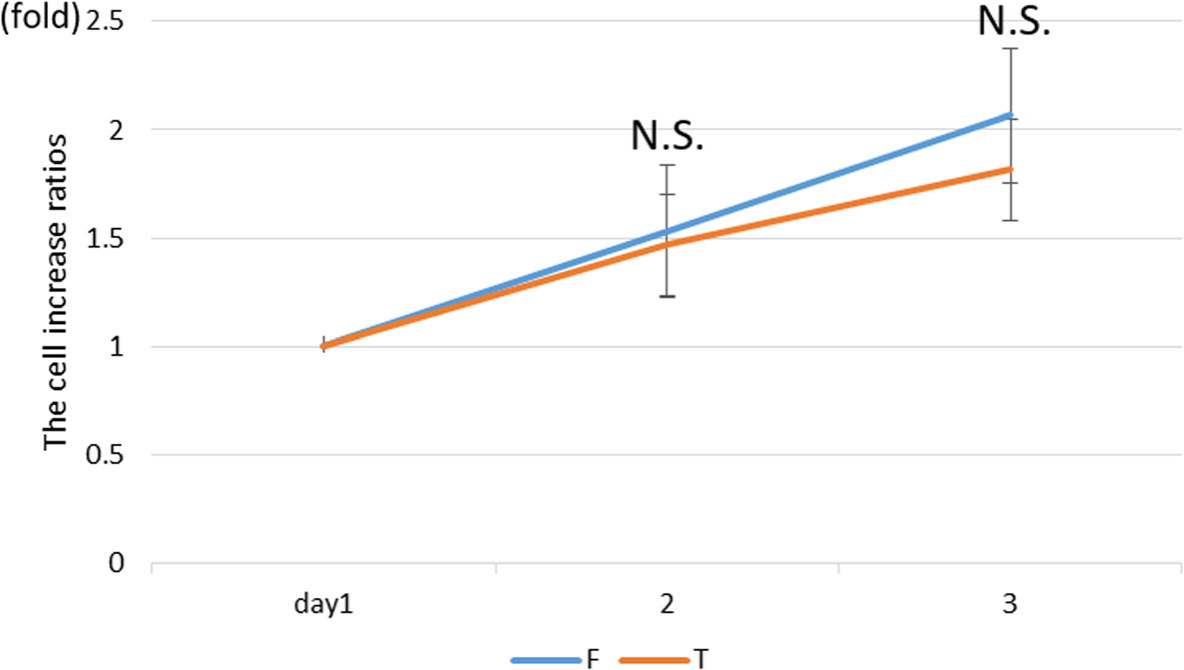
Results of the chondrocyte proliferation ability of isolated knee and ankle articular cartilage. *N.S.* no significant difference

The proliferation rate of chondrocytes was similar over time (Fig. 8). At day 3, chondrocytes of the talus tended to proliferate more than chondrocytes of the distal femur, but there was no significant difference.

### Production of proteoglycans and reaction to IL-1β

**Fig. 9:**
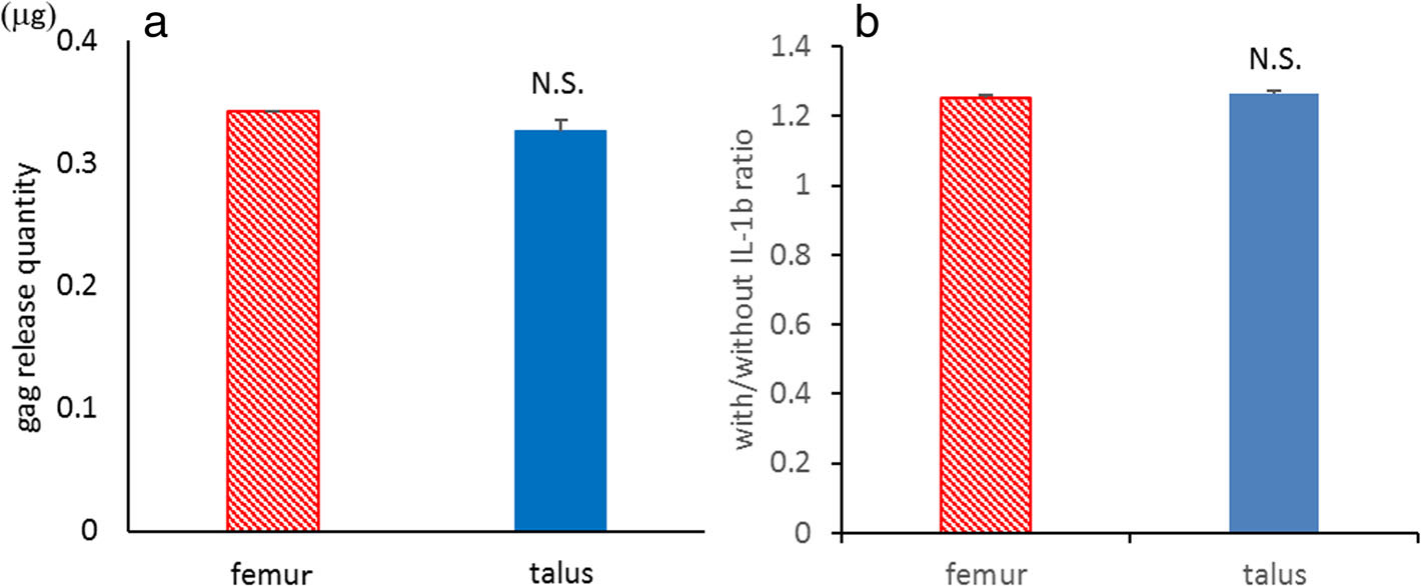
Proteoglycan release quantity (**a**) and reaction to IL-1β (**b**). *N.S.* no significant difference

To determine the quantity of proteoglycan release and the reaction to IL-1β, a proteoglycan release assay for the distal femur and talus with/without IL-1β was performed. There was no significant difference in proteoglycan production (*P* = 0.98) (Fig. 9a) or the reaction to IL-1β between the distal femur and talus (*P* = 0.90) (Fig. 9b).

## Discussion

Our results demonstrate that osteochondral defects in the covered talus, which has good congruency, exhibit early and good repair at 12 weeks after surgery, with the cartilage at the defect appearing similar to the adjacent cartilage. Immunoreactivity of covered talus demonstrated hyaline cartilage in the defect site. The covered talus showed good repair compared to that of the uncovered talus. This indicates that morphological factors may affect osteochondral repair. The covered talus has high congruency, while the uncovered talus does not. Decreased joint congruency results in increased contact pressure per area [21]. Congruent joints can distribute load and reduce the progress of cartilage defects and subchondral cysts. Thus, our results indicate that cartilage repair of congruent joints (covered talus) is faster and better than that of incongruent joints (uncovered talus).

In addition, the improved lesion repair of congruent joints compared to that of incongruent joints relates to subchondral bone repair. Our results indicate that subchondral bone in the osteochondral defect of the covered talus showed early recovery to the level of adjacent bone, which suggests that good cartilage repair may be required for early subchondral bone repair. There is a dynamic relationship between cartilage and subchondral bone, such that an abnormality in either can lead to the loss of balance of the bone cartilage unit. Damaged subchondral bone cannot support the overlying cartilage [22]. Tidemark advancement and thickening of the subchondral plate induced by remodeling of injured subchondral bone are early signs of OA [23]. Shahgaldi et al. reported that contact pressures of reparative articular surfaces were either higher or lower than normal controls and suggested that these differences lead to thickness variations of the surface of repaired tissue and the presence of an abnormal thickness of subchondral plate [24]. Messner [25] and Shapiro et al. [26] postulated that inadequate subsurface support of the subchondral bone bed may be a reason for unsuccessful repair. Thus, the formation of a normal contour of subchondral bone is an important factor for successful osteochondral repair.

Other important factors of osteochondral repair are the characteristics of the cartilage. Several studies have shown differences between the knee and ankle cartilages. Schumacher et al. demonstrated a difference in cell distribution between the knee and ankle cartilages [27]. Horizontal sections of the superficial zone of the ankle contain chondrocytes organized into clusters or chondrons of two to six cells that lie horizontal to the surface. This cell clustering is not observed in knee articular cartilage. In the knee, chondrocytes in the superficial zone exist as single cells or as doublets that are isolated from each other. Chondrocytes in the deep zone of the ankle are observed as either single cells or doublets. Within the knee, 90 % of chondrocytes are present as single cells, whereas only 3.8 % of chondrocytes in the ankle exist as single cells. Chubinskaya et al. and Aurich et al. demonstrated that the GAG content is significantly higher in the ankle than in the knee [28, 29]. The GAG content is reduced in OA cartilage, but this may be due to proteoglycan release from the damaged matrix. In regard to the equilibrium modulus, dynamic stiffness and hydraulic permeability, which define the ability of the extracellular matrix to withstand compressive loads, are higher in the ankle cartilage than in the knee cartilage [30]. Therefore, the ankle cartilage has stiffer cartilage and a greater mean compressive modulus than knee cartilage [31]. The response of ankle chondrocytes to inflammatory molecules is lower than that in the knee because of differences between transport properties within the ankle and knee. Molecules diffuse through avascular cartilage and the rate of diffusion is determined by diffusion and partition coefficients. The diffusion coefficient is similar between the knee and ankle, whereas the partition coefficient is 47 % lower in the ankle than in the knee [32]. Orazizadeh reported a difference of tolerance to biomechanical stress on the cartilage between the knee and ankle. Marked differences in the relative levels of the aggrecan gene mRNA are observed in the response of ankle chondrocytes to 20 min of mechanical stimulation at 0.33 Hz within a sealed pressure chamber compared to the response of knee joint chondrocytes. Ankle chondrocytes are more metabolically active than those of the knee [33]. Deep zone chondrocytes synthesize more PG and collagen than those in the superficial zones in the knee and ankle joints, although there is variation between the different zones within the cartilage [30, 32, 34]. The incidence of OA in the ankle joint is lower than that in the knee joint due to these differences between the knee and ankle cartilages [35, 36]. However, our results showed no significant difference in the proliferation of chondrocytes, the production of PG, or the response to the IL-1β between the knee and ankle cartilages. Thus, our results may reflect differences in joint morphology between the knee and ankle, rather than differences in biochemical properties of cartilage.

There are a few limitations to this study. First, this research did not consider sufficiently the influence of weight bearing. Takahashi et al. reported that loading and unloading in the early phase of cartilage repair has merits and demerits [37]. Yokota et al. reported that loading at a moderate intensity appears to be necessary for cartilage maintenance [38]. In our model, the degree of load varies at each defect site. Therefore, this difference in load on the osteochondral defect site may influence the results of this study. Second, bone marrow cells from the knee and ankle were not evaluated. The talus of the rabbits is very small, and harvesting bone marrow is difficult. Further investigation of differences in bone marrow cells between the knee and ankle is needed. Third, in this study, subchondral bone cysts were not evaluated. Loading compressed cartilage forces its water into subchondral bone that has been drilled, leading to a localized high increased flow and pressure of fluid in the subchondral bone. This results in local osteolysis and development of a subchondral cyst. Subchondral cyst formation is assumed to be caused by the damaged cartilage functioning as a valve [39]. The presence of subchondral bone cysts may affect the results of this study, but obvious subchondral bone cysts were not observed in either histological or radiographical evaluation.

## Conclusions

Our results demonstrate that the covered talus shows early and good osteochondral repair compared to the knee and uncovered talus. These results suggest that the congruent joint has advantages over the non-congruent joint for subchondral repair prior to cartilage repair.
